# Vaccine-Induced, High-Magnitude HIV Env-Specific Antibodies with Fc-Mediated Effector Functions Are Insufficient to Protect Infant Rhesus Macaques against Oral SHIV Infection

**DOI:** 10.1128/msphere.00839-21

**Published:** 2022-02-23

**Authors:** Alan D. Curtis, Pooja T. Saha, Maria Dennis, Stella J. Berendam, Pratamesh Ramasubramanian, Kaitlyn A. Cross, S. Munir Alam, Guido Ferrari, Pamela A. Kozlowski, Genevieve G. Fouda, Michael G. Hudgens, Koen K. A. Van Rompay, Justin Pollara, Sallie R. Permar, Kristina De Paris

**Affiliations:** a Department of Microbiology and Immunology, School of Medicine, University of North Carolina at Chapel Hill, Chapel Hill, North Carolina, USA; b Center for AIDS Research, School of Medicine, University of North Carolina at Chapel Hill, Chapel Hill, North Carolina, USA; c Children’s Research Institute, School of Medicine, University of North Carolina at Chapel Hill, Chapel Hill, North Carolina, USA; d Department of Biostatistics, Gillings School of Public Health, University of North Carolina at Chapel Hill, Chapel Hill, North Carolina, USA; e Duke Human Vaccine Institute, Duke University Medical Center, Durham, North Carolina, USA; f Department of Surgery, Duke University School of Medicine, Durham, North Carolina, USA; g Department of Molecular Genetics and Microbiology, Duke University Medical Center, Durham, North Carolina, USA; h Department of Microbiology, Immunology and Parasitology, Louisiana State University Health Sciences Center, New Orleans, Louisiana, USA; i California National Primate Research Center, University of California at Davis, Davis, California, USA; j Department of Pediatrics, Weill Cornell Medical College, New York, New York, USA; University of Florida

**Keywords:** ADCC, Fc-mediated antibody function, pediatric HIV vaccine, rhesus macaque

## Abstract

Improved access to antiretroviral therapy (ART) and antenatal care has significantly reduced *in utero* and peripartum mother-to-child human immunodeficiency virus (HIV) transmission. However, as breast milk transmission of HIV still occurs at an unacceptable rate, there remains a need to develop an effective vaccine for the pediatric population. Previously, we compared different HIV vaccine strategies, intervals, and adjuvants in infant rhesus macaques to optimize the induction of HIV envelope (Env)-specific antibodies with Fc-mediated effector function. In this study, we tested the efficacy of an optimized vaccine regimen against oral simian-human immunodeficiency virus (SHIV) acquisition in infant macaques. Twelve animals were immunized with 1086.c gp120 protein adjuvanted with 3M-052 in stable emulsion and modified vaccinia Ankara (MVA) virus expressing 1086.c HIV Env. Twelve control animals were immunized with empty MVA. The vaccine prime was given within 10 days of birth, with booster doses being administered at weeks 6 and 12. The vaccine regimen induced Env-specific plasma IgG antibodies capable of antibody-dependent cellular cytotoxicity (ADCC) and phagocytosis (ADCP). Beginning at week 15, infants were exposed orally to escalating doses of heterologous SHIV-1157(QNE)Y173H once a week until infected. Despite the induction of strong Fc-mediated antibody responses, the vaccine regimen did not reduce the risk of infection or time to acquisition compared to controls. However, among vaccinated animals, ADCC postvaccination and postinfection was associated with reduced peak viremia. Thus, nonneutralizing Env-specific antibodies with Fc effector function elicited by this vaccine regimen were insufficient for protection against heterologous oral SHIV infection shortly after the final immunization but may have contributed to control of viremia.

**IMPORTANCE** Women of childbearing age are three times more likely to contract HIV infection than their male counterparts. Poor HIV testing rates coupled with low adherence to antiretroviral therapy (ART) result in a high risk of mother-to-infant HIV transmission, especially during the breastfeeding period. A preventative vaccine could curb pediatric HIV infections, reduce potential health sequalae, and prevent the need for lifelong ART in this population. The results of the current study imply that the HIV Env-specific IgG antibodies elicited by this candidate vaccine regimen, despite a high magnitude of Fc-mediated effector function but a lack of neutralizing antibodies and polyfunctional T cell responses, were insufficient to protect infant rhesus macaques against oral virus acquisition.

## INTRODUCTION

The successful implementation of antiretroviral therapy (ART) for women living with human immunodeficiency virus (HIV) has resulted in a drastic reduction of *in utero* and peripartum mother-to-child transmission of HIV type 1 (HIV-1) in the last 2 decades. Yet globally, between 400 and 500 infants continue to acquire HIV every day (1). The majority of these infections occur during the breastfeeding period. Limited access to ART in rural communities, HIV diagnosis late in pregnancy, gaps in linking antenatal care with postnatal mother and infant care, acute maternal infection during the breastfeeding period, and lack of ART adherence impede the prevention of HIV transmission by breast milk (2–9). Transmission of HIV can occur throughout the breastfeeding period, with a cumulative risk increase with every month of breastfeeding (10–13). However, in many resource-limited countries, breast milk remains a necessary choice for nutrition and to provide passive immunity to protect the infant against other endemic pathogens (6, 7, 14). Indeed, early weaning is associated with increased infant mortality (15–17), and the WHO recommends exclusive breastfeeding for 6 to 12 months for infants born to HIV-infected mothers (18). Infants born to mothers with known HIV-positive status are tested at birth and immediately started on ART if found to be infected, whereas infants who acquire HIV by breastfeeding often go undiagnosed until they develop clinical symptoms. Prolonged HIV replication prior to diagnosis may severely interfere with multiple aspects of normal immune and central nervous system development and impede immune reconstitution after ART initiation. Therefore, prevention strategies tailored to infants are needed to further reduce the risk of pediatric HIV infections.

In nonhuman primate (NHP) models of HIV, infection of neonatal and infant rhesus macaques (RM) with simian-human immunodeficiency virus (SHIV) can be prevented by passive administration of broadly neutralizing HIV envelope (Env)-specific antibodies (bNAbs) (19–21). The use of bNAbs as potential prevention strategy in HIV-exposed infants is supported by results from ongoing clinical trials that indicate that bNAbs (e.g., VRC01) are safe and well tolerated in human neonates (22). Clinical studies in human adults, however, demonstrated only a minimal risk reduction of HIV infection by preventative treatment with bNAbs (23, 24). Therefore, the development of an effective HIV vaccine remains a high priority for this risk group. While the induction of HIV bNAbs by vaccination remains challenging, antibodies with Fc-mediated effector function can be induced more consistently and have been associated with partial protection in multiple NHP vaccine/challenge studies (25–29) and in the human RV144 HIV vaccine trial (30). Furthermore, the protective effect of bNAbs is not due solely to their neutralization function but also depends, at least in part, on the Fc-mediated effector functions of these bNAbs (31, 32).

Utilizing the pediatric rhesus macaque model, we previously compared different HIV vaccine modalities, immunization intervals, and adjuvants to optimize the induction of HIV Env-specific IgG antibodies with Fc-mediated effector functions (33–35). Building on these results, in the current study, we tested the efficacy of an intramuscular (i.m.) vaccine consisting of a modified vaccinia Ankara (MVA) virus vector expressing transmitted/founder virus 1086.c gp120 combined with 1086.c HIV gp120 protein and 3M-052 adjuvant in stable emulsion against oral SHIV acquisition in infant macaques. Consistent with our prior findings, the vaccine induced high-magnitude Env-specific antibodies in plasma with potent antibody-dependent cellular cytotoxicity (ADCC) and antibody-dependent cellular phagocytic (ADCP) function. Nonetheless, these responses did not protect infant rhesus macaques against subsequent heterologous oral SHIV challenge.

## RESULTS

### Study design.

**FIG 1:**
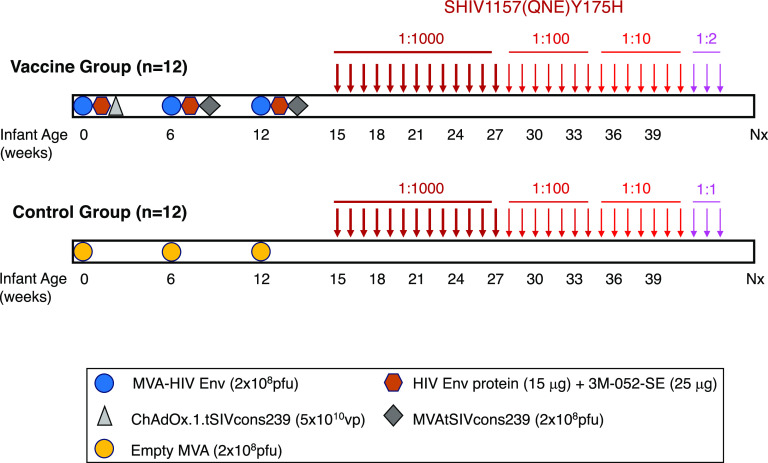
Experimental design. In the vaccine group, 12 neonatal rhesus macaques (Table 1) were immunized with 2 × 10^8^ PFU of MVA-HIV Env, HIV Env protein (15 μg) mixed with 3M-052-SE, and 5 × 10^10^ ChAdOx1.tSIVconsv239 viral particles at week 0. Booster immunizations of 2 × 10^8^ PFU each of MVA-HIV Env, HIV Env protein in 3M-052-SE, and MVA.tSIVconsv239 were provided at weeks 6 and 12. A second cohort of 12 age-matched RM received control MVA immunizations at weeks 0, 6, and 12. Beginning at week 15, animals were challenged weekly with SHIV-1157(QNE)Y173H viral stock diluted 1:1,000 in RPMI 1640 medium until infected. After 13 exposures, uninfected infants (*n* = 11) were exposed to a 1:100 SHIV dose for 7 weeks, a dose that was increased to 1:10 for seven more exposures in animals not infected by the 1:100 dose (*n* = 4). Two infants remained negative and became infected after challenge with 1:2 dilution of virus stock (RM19) or undiluted (1:1) virus (RM10) (Table 1). SHIV exposures are indicated by arrows with distinct shades of red based on virus dilution.

The current study utilized infant RM that were randomly divided into 2 groups of 12 at birth (Table 1; Fig. 1). Infant RM in the vaccine group were immunized at week 0 with 2 × 10^8^ PFU of MVA-HIV 1086.c Env construct and 15 μg of 1086.c gp120 protein mixed with 3M-052-SE adjuvant by the i.m. route. At weeks 6 and 12, infants in the vaccine cohort received i.m. booster immunizations with MVA-HIV Env and 1086.c gp120 protein in 3M-052-SE. In addition, to induce simian immunodeficiency virus (SIV)-specific T cell responses, infant vaccinees were primed with 5 × 10^10^ viral particles of ChAdOx1.tSIVconsv239 expressing conserved SIV Gag/Pol epitopes at week 0. These responses were boosted by immunizations with 2 × 10^8^ PFU of MVA.tSIVconsv239 at weeks 6 and 12. Control infants received an empty MVA vector at weeks 0, 6, and 12 (Fig. 1). Once-weekly oral SHIV challenges were initiated at week 15, 3 weeks after the last immunization. Animals were followed for approximately 12 weeks post-SHIV infection, with infection being defined two consecutive positive viral RNA results for an animal.

**TABLE 1:** Summary of study animals

Group	Animal	Sex	Age (days) at 1st immunization	No. of challenges to achieve infection	Infecting dose	Peak viremia (copies/mL)
Mock	RM1	Female	8	17	1:100	5.1 × 10^7^
Mock	RM2	Male	8	3	1:1,000	1.3 × 10^8^
Mock	RM3	Male	6	13	1:1,000	2.5 × 10^6^
Mock	RM4	Female	6	14	1:100	7.1 × 10^5^
Mock	RM5	Male	5	7	1:1,000	7.6 × 10^6^
Mock	RM6	Female	4	4	1:1,000	1.3 × 10^8^
Mock	RM7	Female	10	8	1:1,000	8.9 × 10^6^
Mock	RM8	Male	10	15	1:100	4.3 × 10^7^
Mock	RM9	Female	7	2	1:1,000	2.1 × 10^7^
Mock	RM10	Male	7	30	Undiluted	3.1 × 10^4^
Mock	RM11	Male	6	2	1:1,000	1.6 × 10^7^
Mock	RM12	Male	4	3	1:1,000	3.5 × 10^8^
Vaccine	RM13	Female	8	3	1:1,000	5.3 × 10^6^
Vaccine	RM14	Male	7	15	1:100	1.7 × 10^7^
Vaccine	RM15	Male	5	24	1:10	5.1 × 10^5^
Vaccine	RM16	Male	5	1	1:1,000	2.0 × 10^6^
Vaccine	RM17	Male	4	4	1:1,000	1.1 × 10^6^
Vaccine	RM18	Male	3	2	1:1,000	1.2 × 10^7^
Vaccine	RM19	Female	8	28	1:2	9.7 × 10^6^
Vaccine	RM20	Male	8	3	1:1,000	4.7 × 10^6^
Vaccine	RM21	Female	7	7	1:1,000	2.1 × 10^6^
Vaccine	RM22	Male	7	15	1:100	4.5 × 10^7^
Vaccine	RM23	Female	6	20	1:100	5.3 × 10^7^
Vaccine	RM24	Male	6	23	1:10	1.4 × 10^7^

### Vaccine-induced 1086.c envelope-specific antibody responses.

**FIG 2:**
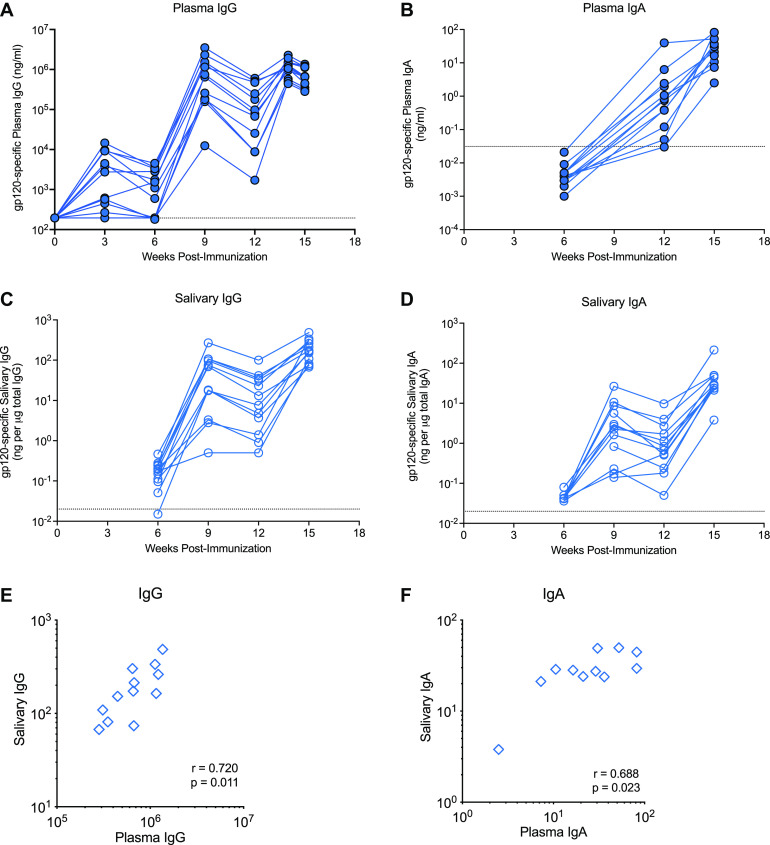
C.1086 Env-specific antibody responses. Plasma concentrations of 1086.c gp120-specific IgG (A) and IgA (B) were measured by ELISA and BAMA, respectively. Salivary IgG and IgA levels, measured by BAMA, are reported as specific activity in nanograms of 1086.c gp120 IgG or IgA per μg of total IgG (C) or IgA (D). Dashed lines represent the cutoff for positivity, defined as mean antibody levels in control animals plus 3 standard deviations (SD). Panels E and F illustrate the Spearman correlation between plasma and saliva vaccine-induced IgG and IgA levels, respectively. Each symbol represents an individual animal of the 12 vaccinated animals.

We first aimed to confirm our prior findings that the vaccine regimen induces potent HIV Env-specific antibody responses (35). Plasma 1086.c gp120-specific IgG responses were detected as early as week 3 after the first immunization in the majority of animals (Fig. 2A). Antibody levels were enhanced following the week 6 booster immunization, waned slightly thereafter, and reached peak levels after the final immunization at week 12. Geometric mean plasma HIV Env-specific IgG concentrations at week 14 (1,060,401 ng/mL; 95% confidence interval [CI], 1,470,184; 21,020) were comparable to those elicited in our prior study (1,251,467 ng/mL; 95% CI, 1,049,651; 53,481) (35). We also tested for the induction of Env-specific plasma IgA antibody in vaccinated infants (Fig. 2B). The induction of plasma Env-specific plasma IgA was delayed compared to that of plasma IgG and was of lower magnitude. Env-specific IgG and IgA were also detectable in saliva (Fig. 2C and D). The positive correlation between plasma and salivary Env-specific IgG and IgA (Fig. 2E and F) implied that antibodies in saliva likely reflected transudation from the plasma rather than local induction at mucosal sites. The limited saliva volumes did not allow us to test for the secretory component of IgA to determine mucosal antibody production.

**FIG 3:**
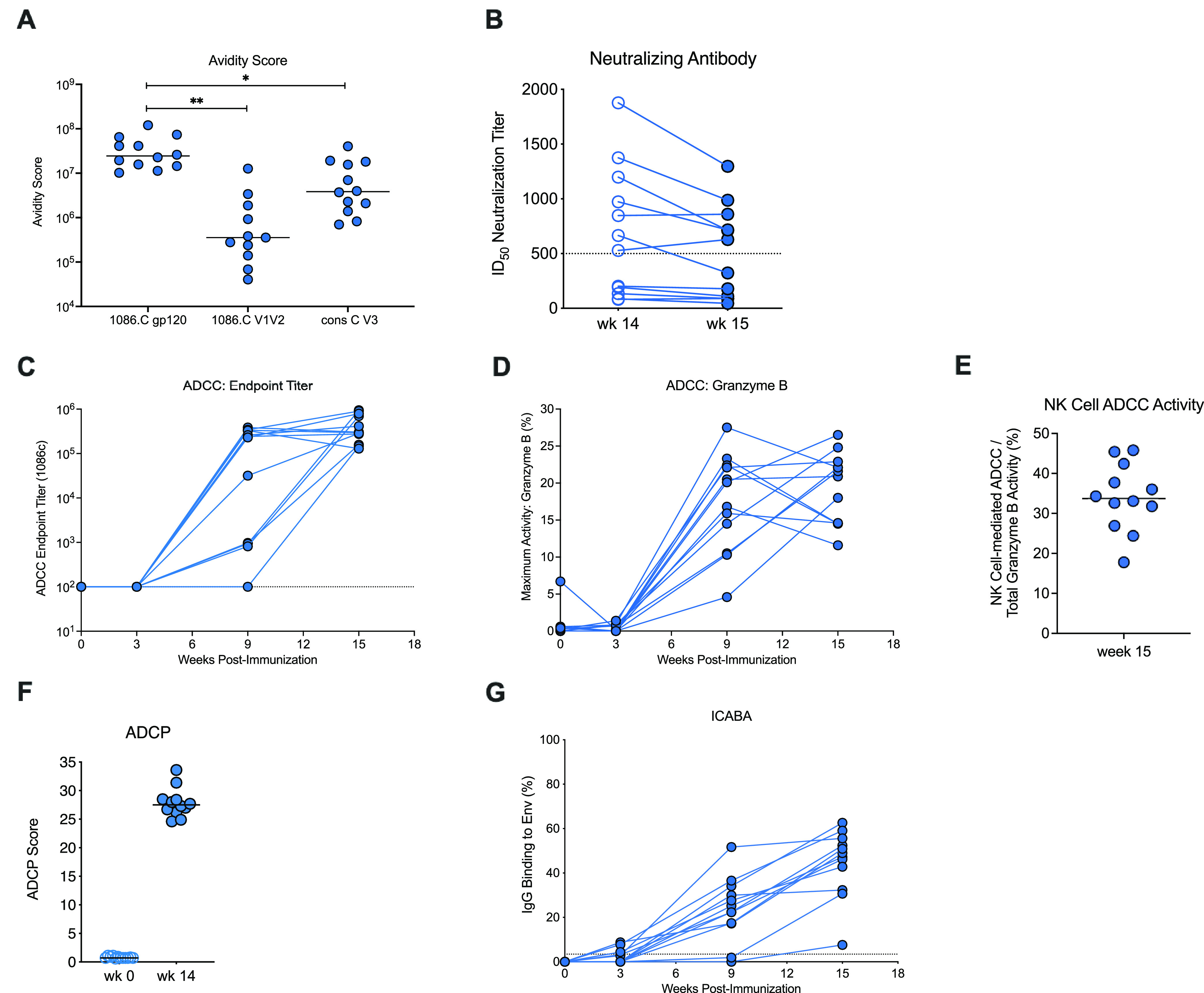
Prechallenge antibody function of vaccinated infant macaques. (A) Avidity score, determined by SPR, of week 15 plasma IgG specific for 1086.c gp120 or V1V2 or for the consensus clade C V3 (gp70). Each symbol represents a single animal. Note that only 11 of 12 animals were included in the testing for 1086.c V1V2 avidity due to limited plasma volumes. (B) Tier 1b clade C I6644.v2.c33 neutralization titers of vaccinated infants at week 14 and week 15. (C and D) Longitudinal data for ADCC endpoint titers and maximum granzyme B activity, with each line representing an individual animal. Dashed lines indicate the limit of detection. (E) The percentage of monocyte-independent, NK cell-mediated ADCC activity at week 15. (F) ADCP scores for vaccinated animals prior to vaccination at week 0 and week 14. (G) Plasma IgG binding to cells infected with HIV 1086.c is shown over time for individual vaccinated animals. Each time point shows data for all 12 of the vaccinated animals if not indicated otherwise.

We next evaluated the avidity and functional potential of Env-specific plasma IgG. The avidity of plasma IgG specific for 1086.c gp120 was measured by surface plasmon resonance (SPR), and the median avidity score at week 14 was determined to be 2.4 × 10^7^ (95% CI, 1.45 × 10^7^, 6.5 × 10^7^) (Fig. 3A), an avidity similar (*P* = 0.4; Wilcoxon rank sum test) to the one in our previous study (median avidity score, 4.6 × 10^7^; 95% CI, 1.2 × 10^7^, 9.6 × 10^7^) (35). The avidity of plasma vaccine-elicited IgG was stronger for the clade C consensus V3 than for the V1V2 epitope of 1086.c Env (Fig. 3A). The current vaccine regimen elicited weak clade C tier 1 neutralization antibodies. In 7 of 12 vaccinated infants, the peak neutralization titers against the tier 1b virus I6644.v2.c33 were >500 at week 14, but only 5 of the 7 animals had maintained tier 1b 50% inhibitory dilution (ID_50_) titers of >500 by week 15 (Fig. 3B).

Because a main goal of the current design was to elicit nonneutralizing antibody, we assessed the propensity of vaccine-elicited plasma antibody for FcR-mediated ADCC. ADCC responses against Env 1086.c gp120 were detectable in 75% of vaccinated infants by week 9 and in 100% by the time of initial SHIV challenge at week 15 (Fig. 3C). Similar to vaccine-induced plasma Env-specific IgG, the high ADCC endpoint titers and median granzyme B activity (Fig. 3D) in the current study were comparable to those observed in our prior study (35). To assess the ADCC activity that was independent of monocytes and could be attributed exclusively to NK cells (36), we performed area scaling (37). NK cell-mediated ADCC activity ranged from 17.8% to 45.8% (median, 33.75 [Fig. 3E]). In addition to ADCC, 1086.c Env-specific plasma antibodies were also able to mediate ADCP (Fig. 3F). Relevant to both ADCC and ADCP function, plasma IgG was capable of binding to Env 1086.c expressed on the surface of HIV-infected cells (ICABA), with 11 of 12 infants having >20% binding at week 15 (range, 7.60% to 62.62%; mean, 44.70%) (Fig. 3G).

**FIG 4:**
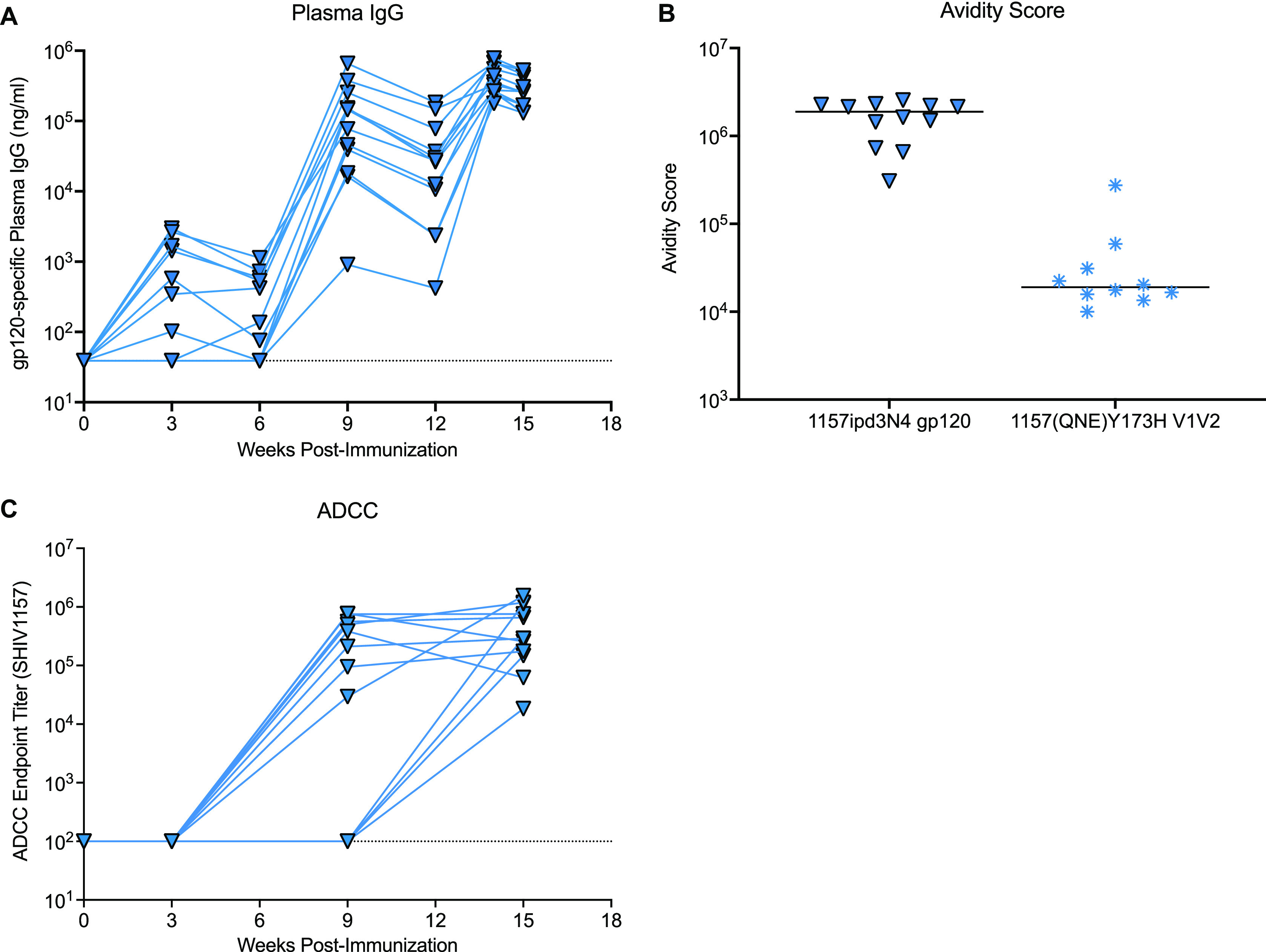
Vaccine-induced 1157ipd3N4 and SHIV1157(QNE)Y175H Env-specific antibody responses. (A) Plasma concentration of 1157ipd3N4 gp120-specific IgG over time in the 12 vaccinated infant rhesus macaques. (B) Avidity scores of plasma IgG specific for 1157ipd3N4 gp120 (*n* = 12) or gp70-V1V2 SHIV1157(QNE)Y375H (*n* = 10). Each symbol represents an individual animal; horizontal lines represent the medians. Note that only 10 animals could be tested for the avidity of antibodies to gp70-V1V2 SHIV1157(QNE)Y375H due to the limited plasma volumes available from infant rhesus macaques. (C) ADCC endpoint titers for plasma antibodies specific to 1157ipd3N4 gp120 in the 12 vaccinated animals.

We also measured antibody responses relevant to the heterologous SHIV challenge virus, including clade C 1157ipd3N4 Env-specific IgG and 1157(QNE)Y173H Env V1V2-specific antibody responses. Although the overall magnitude of plasma binding antibodies to 1157ipd3N4 gp120 was lower than for 1086.c gp120-specific IgG, the kinetics of plasma binding antibodies to 1157ipd3N4 Env followed a pattern similar to that observed for 1086.c gp120-specific IgG. All animals developed 1157ipd3N4 gp120-specific IgG after the second immunization, with peak responses at week 14, 2 weeks after the third immunization (Fig. 4A). The median avidity score of plasma IgG against 1157id3N4 gp120 (1.9 × 10^6^; 95% CI, 7.3 × 10^6^, 2.3 × 10^6^]) was about 1 log lower than the avidity index for the vaccine immunogen 1086.c gp120, and the avidity for the V1V2 region of 1157(QNE)Y173H was 1 log lower than the avidity for 1086.c V1V2 (Fig. 3A and 4B). The vaccine regimen elicited high levels of Env-specific plasma antibodies with ADCC activity against the clade C 1157ipd3N4 gp120 (Fig. 4C), with median endpoint titers (2.89 × 10^5^) comparable to the median titer for 1086.c (2.99 × 10^5^ [Fig. 3B]), although ADCC 1157ipd3N4-specific IgG endpoint titers exhibited greater variability among the individual animals.

### Cellular responses to vaccination.

**FIG 5:**
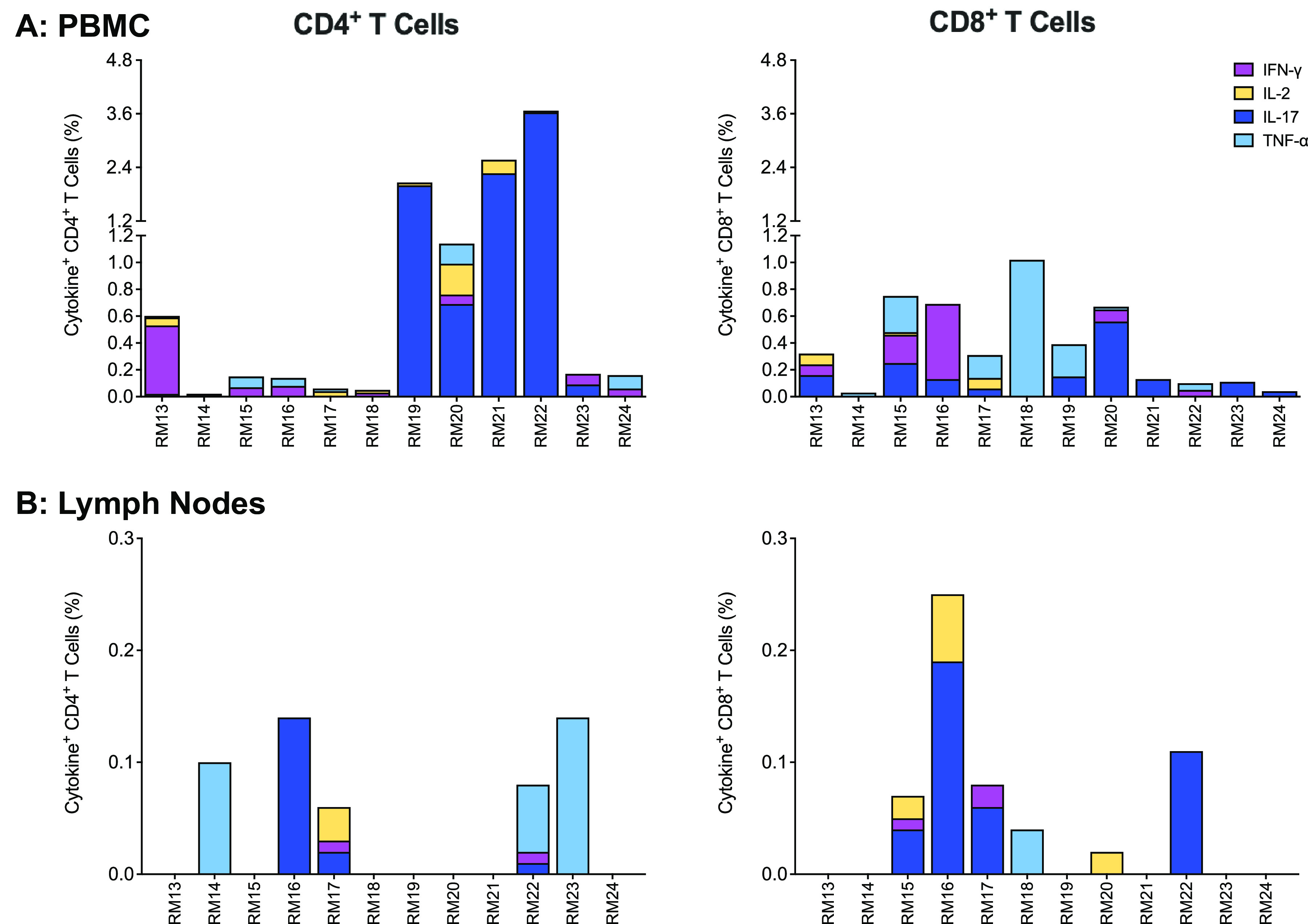
SIV Gag-specific T cell responses in PBMCs (*n* = 12) and peripheral lymph nodes (*n* = 12) at week 14. Each bar in panels A and B represents the sum of single cytokine responses of SIV Gag-specific CD4^+^ (left graphs) or CD8^+^ T cells (right graphs) for each vaccinated animal at week 14 in PBMCs (A) or lymph nodes (B). Cytokines measured include gamma interferon (IFN-γ), IL-2, IL-17, and TNF-α.

The majority of vaccinated animals developed SIV Gag-specific T cell responses by week 14 in peripheral blood (Fig. 5A). In lymph nodes, SIV Gag-specific T cell responses were detected in 9 of 12 vaccinees. (Fig. 5B). SIV Gag-specific CD4^+^ T cells appeared to produce predominantly tumor necrosis factor alpha (TNF-α) and interleukin 17 (IL-17), whereas a more mixed cytokine response was observed in CD8^+^ T cells. Polyfunctional cytokine responses were rare.

### SHIV1157(QNE)Y173H challenge outcome.

**FIG 6:**
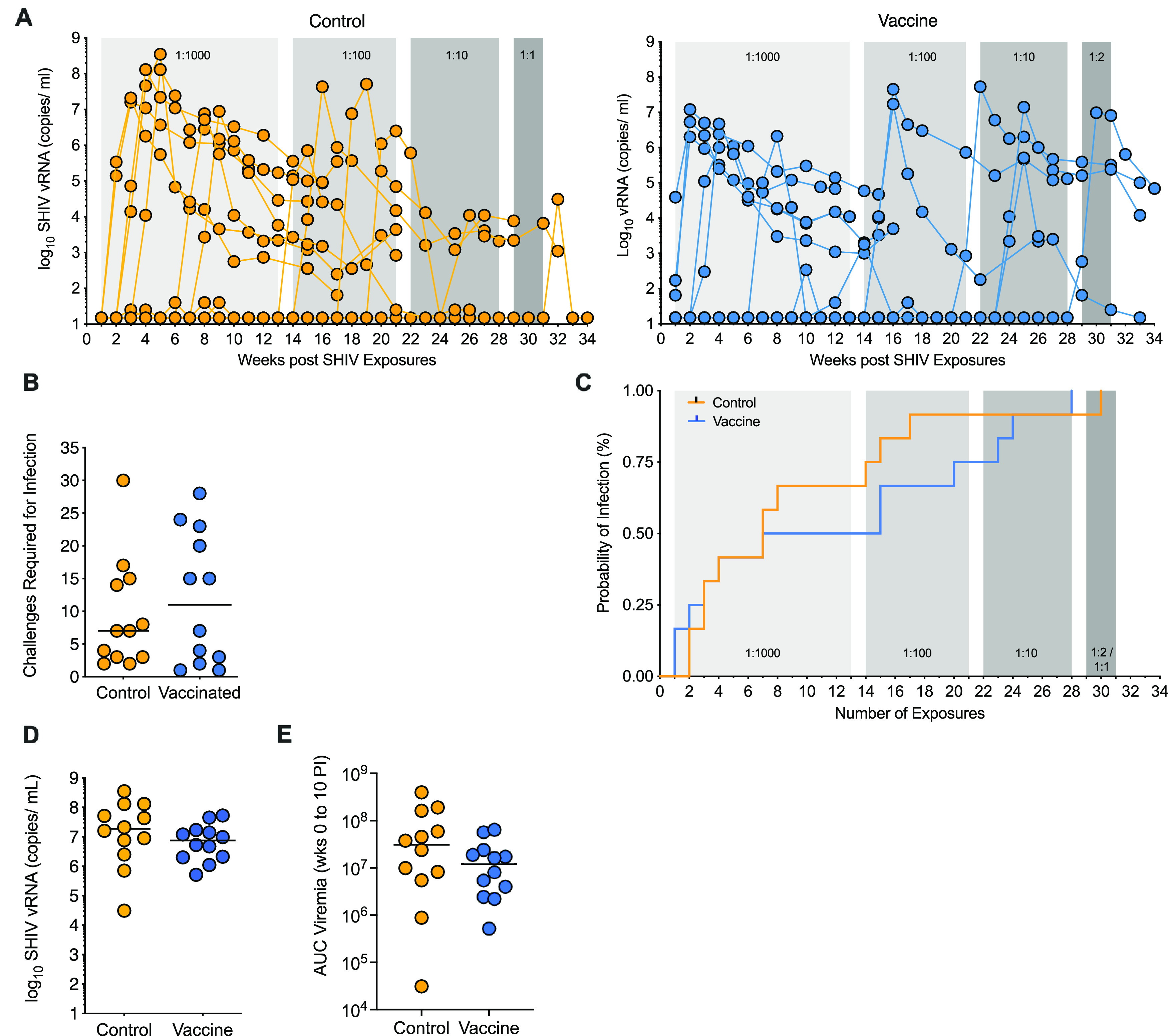
Challenge outcome. (A) Longitudinal plasma viral load measurements as assessed by RT-PCR from control (*n* = 12) and vaccinated (*n* = 12) cohorts of infant RM are displayed in copies per milliliter plasma. Shaded areas represent the challenge doses: light gray, 1:000, weeks 0 to 13; medium gray, 1:100, weeks 14 to 21; dark gray, 1:10, weeks 22 to 28; darkest gray, 1:2 or undiluted. (B) The number of challenges required for infection is plotted for control (*n* = 12) and vaccinated (*n* = 12) animals. Horizontal lines represent the medians. (C) Kaplan-Meier survival curves for any dose of viral stock dilutions are shown for control and vaccinated infants. (D and E) Peak viremia (D) and area-under-the curve (AUC) viremia from weeks 0 to 10 postinfection (PI) (E) in control (*n* = 12) and vaccinated (*n* = 12) animals. Control and vaccinated animals are indicated by orange or blue lines/symbols, respectively, with each symbol representing an individual animal; horizontal lines indicate the medians.

Starting at week 15, 3 weeks after the third immunization, animals were challenged once weekly with SHIV by the oral route. The initial virus dose consisted of 1:1,000-diluted virus stock, a dose that was purposely chosen to be 10-fold higher than the dose (1:10,000) successfully used in an intrarectal (i.r.) challenge study in adult rhesus macaques (28), because of the lower risk estimate for oral versus i.r. infection determined by human HIV epidemiologic studies (38) and SHIV1157 infections in adult RM (39). Seven of 12 control vaccinated infants became infected at the 1:1,000 SHIV dose, and 4 of the remaining 5 animals became infected at 1:100. RM10 remained uninfected after 29 challenges and became infected only after oral challenge with undiluted viral stock (Fig. 6A; Table 1). The median challenge number required to become infected for control vaccinated infants was 7.5. In comparison, vaccinated animals required a median number of 11 challenges to achieve infection (Fig. 6B). Half of the vaccinated animals (*n* = 6) were infected at the 1:1,000 dose and three additional animals at the 1:100 dose. The remaining 3 animals were infected by 1:10 (*n* = 2) and 1:2 (*n* = 1) challenge virus dilutions (Fig. 6A). Although vaccinated animals required a slightly higher average number of challenges to achieve infection (11 exposures) compared to controls (7.5 exposures), there was no difference in the probability of infection at any challenge dose between the two groups (*P* = 0.89) (Fig. 6C). When we compared the probabilities of infection between control and vaccinated animals that became infected at the 1:1,000 challenge virus dose, at the 1:1,000 or 1:100 dose, or at the 1:1,000, 1:100, or 1:10 dose, we also did not detect differences in infection risks. The distributions of peak viremia also did not differ between vaccinated animals (median, 1.85 × 10^7^ viral RNA copies/mL) and control animals (median, 7.5 × 10^6^ viral RNA copies/mL; Wilcoxon rank sum test with exact *P* value of 0.24) (Fig. 6D). Similarly, there was no difference found when we compared area-under-the curve viremia from week 0 to week 10 postinfection between the two groups (*P* = 0.1978) (Fig. 6E).

### Immune correlates of challenge outcome.

**FIG 7:**
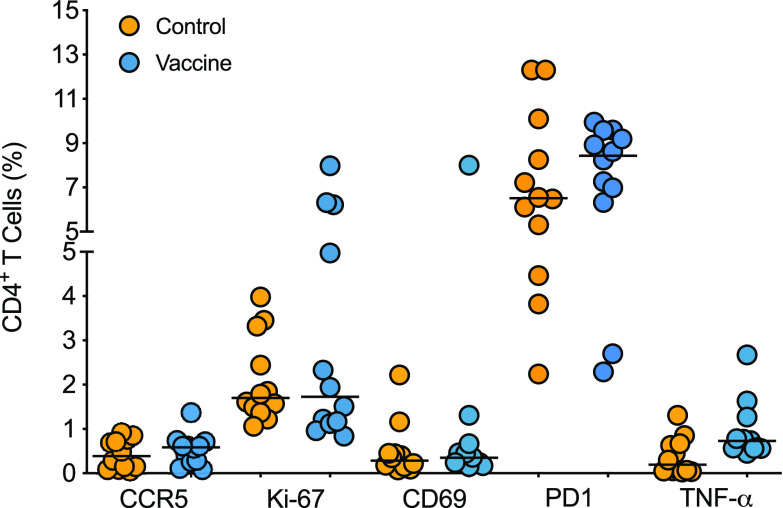
CD4^+^ T cell activation. PBMCs from week 15 after vaccination were gated on CD3^+^ CD4^+^ T cells and assessed for surface expression of CD195 (CCR5), CD69, and CD279 (PD1) and intracellular expression of Ki-67 and TNF-α. TNF-α-positive T cell frequencies between control (*n* = 12) and vaccinated (*n* = 12) animals were compared by Mann-Whitney test.

To rule out that the vaccine had caused nonspecific immune activation that could promote increased susceptibility to infection (40, 41), we tested for activation of peripheral blood CD4^+^ T cells, the main target cells for HIV. At the time of challenge initiation (week 15), we noted no difference in the frequency distributions of CCR5^+^ (CD195^+^), Ki-67^+^, CD69^+^, or CD279^+^ (PD1) CD4^+^ T cells in blood of vaccinated compared to control animals (Fig. 7). Although vaccinated animals had greater median frequencies of PD-1-positive and TNF-α-producing CD4^+^ T cells than the control group (Fig. 7), there was no correlation with this response and the number of exposures required to achieve infection (Table 2).

**TABLE 2:** Correlation between prechallenge immune parameters and number of challenges required for infection

Parameter	*n*	Spearman *r* value	Correlation *P* value^*c*^	FDR p-value	Figure
T cell activation^*a*^					
CCR5^+^ CD4^+^	24	−0.065	0.7631	0.7648	Fig. 7
Ki-67^+^ CD4^+^	24	−0.161	0.4499	0.6748	Fig. 7
CD69^+^ CD4^+^	24	0.379	0.0688	0.4131	Fig. 7
PD1^+^ CD4^+^	24	−0.064	0.7648	0.7648	Fig. 7
TNF-α^+^ CD4^+^	24	−0.182	0.3913	0.6748	Fig. 7
Env-specific B cells^*b*^	12	−0.336	0.2845	0.6748	
Env-specific IgG^*b*^					
1086.c	12	−0.519	0.0864	0.4548	Fig. 2A
1157ipd3N4	12	−0.470	0.1246	0.4548	Fig. 4A
Plasma IgM^*b*^	11	−0.288	0.3885	0.6347	
Salivary IgG^*b*^	12	−0.456	0.1373	0.4548	Fig. 2C
Salivary IgA^*b*^	12	−0.189	0.5516	0.6818	
Avidity index^*b*^					
1086.c	12	−0.368	0.2365	0.5122	Fig. 3A
1157ipd3N4	12	−0.165	0.6064	0.6818	Fig. 4B
SHIV1157(QNE)Y175H	10	−0.372	0.2880	0.5190	Fig. 4B
1086.C V1V2	11	−0.119	0.7282	0.7706	Fig. 3A
gp70 ConsC V3	12	−0.428	0.1655	0.4548	Fig. 3A
Tier 1 NAbs^*b*^^,^^*d*^	12	0.023	0.9448	0.9448	
ADCC titer^*b*^					
1086.c	12	**−0.761** ^*e*^	**0.0054**	0.0984	Fig. 3C
1157ipd3N4	12	−0.568	0.0580	0.4548	Fig. 4C
ADCC activity^*b*^					
1086.c	12	−0.354	0.2561	0.5122	Fig. 3D
1157ipd3N4	12	−0.418	0.1769	0.4548	
NK cell-mediated ADCC activity^*b*^					
1086.c	12	−0.176	0.5829	0.6818	Fig. 3E
ADCP score^*b*^	12	−0.193	0.5455	0.6818	Fig. 3F
Infected cell binding^*b*^	12	0.242	0.4446	0.6677	Fig. 3G

a Exact *p*–value to test whether the correlation appears to be significantly different from 0.

b FDR adjustment for multiple comparisons for the statistical analyses of cellular correlates of protection.

c FDR adjustment for multiple comparisons for the statistical analyses of antibody correlates of protection.

d ID_50_ neutralizing antibody titers at week 15.

e Bold values indicate Spearman rank test with *r* > 0.6 and unadjusted *p* < 0.05.

Despite the lack of protection against infection, we assessed whether vaccine-induced antibody responses at week 15 were associated with the number of challenges required to achieve infection (Table 2) or with peak viremia (Table 3). Although plasma IgG concentrations specific for 1086.c or 1157ipd3N4 Env were not associated with the number of exposures required to achieve infection (Table 2), there was a negative correlation between 1086.c Env-specific plasma IgG levels and peak viremia (*r* = −0.657, unadjusted *P* = 0.0238, and false-discovery-rate [FDR]-adjusted *P* = 0.1426). There was also a trend toward a negative association of salivary 1086.c Env-specific IgG (*r* = −0.517, unadjusted *P* = 0.0888, and FDR-adjusted *P* = 0.3561) and SHIV1157ipd3N4 Env-plasma IgG concentrations (*r* = −0.0504, unadjusted *P* = 0.0989, and FDR adjusted *P* = 0.3561) with peak viremia at week 15 (Table 3). We did not detect associations between plasma IgG avidity and challenge outcome. Consistent with their low titers at week 15, tier 1 neutralizing antibodies were not associated with the number of exposures required for infection or with peak viremia (Tables 2 and 3).

**TABLE 3:** Correlation between vaccine-induced antibody responses at week 15 and peak viremia

Parameter	*n*	Spearman *r* value	Correlation p value^*b*^	FDR^*a*^ p-value	Figure
Env-specific IgG^*a*^					
1086.c	12	**−0.657** ^*c*^	**0.0238**	0.1426	Fig. 2A
1157ipd3N4	12	−0.504	0.0989	0.3561	Fig. 4A
Plasma IgM^*a*^	11	−0.282	0.4023	0.7761	
Salivary IgG^*a*^	12	−0.517	0.0888	0.3561	Fig. 2C
Salivary IgA^*a*^	12	−0.112	0.7329	0.7761	
Avidity index^*a*^					
1086.c	12	−0.168	0.6039	0.7761	Fig. 3A
1157ipd3N4	12	0.196	0.5431	0.7761	Fig. 4B
SHIV1157(QNE)Y175H	10	−0.152	0.6821	0.7761	Fig. 4B
1086.C V1V2	11	−0.064	0.8603	0.8603	Fig. 3A
gp70 ConsC V3	12	−0.245	0.4434	0.7761	Fig. 3A
Tier 1 NAbs^*d*^	12	−0.112	0.7277	0.7761	
ADCC titer^*a*^					
1086.c	12	−0.238	0.4572	0.7761	Fig. 3C
1157ipd3N4	12	−0.196	0.5431	0.7761	Fig. 4C
ADCC activity^*a*^					
1086.c	12	−0.375	0.2280	0.6839	Fig. 3D
1157ipd3N4	12	−0.217	0.4990	0.7761	
NK cell-mediated ADCC^*a*^					
1086.c	12	**−0.734**	**0.0087**	0.1163	Fig. 8B
ADCP score^*a*^	12	**0.706**	**0.0129**	0.1163	Fig. 3F
Infected cell binding^*a*^	12	−0.147	0.6509	0.7761	Fig. 3G

a FDR adjustment for multiple comparisons for the sets of tests specified in Materials and Methods for the statistical analysis of antibody correlates of protection.

b Exact *p*-value to test whether the correlation appears to be significantly different from 0.

c Bold values indicate Spearman rank correlations with *r* > −0.6 and unadjusted *p* < 0.05.

d ID_50_ neutralizing antibody titers at week 15.

**FIG 8:**
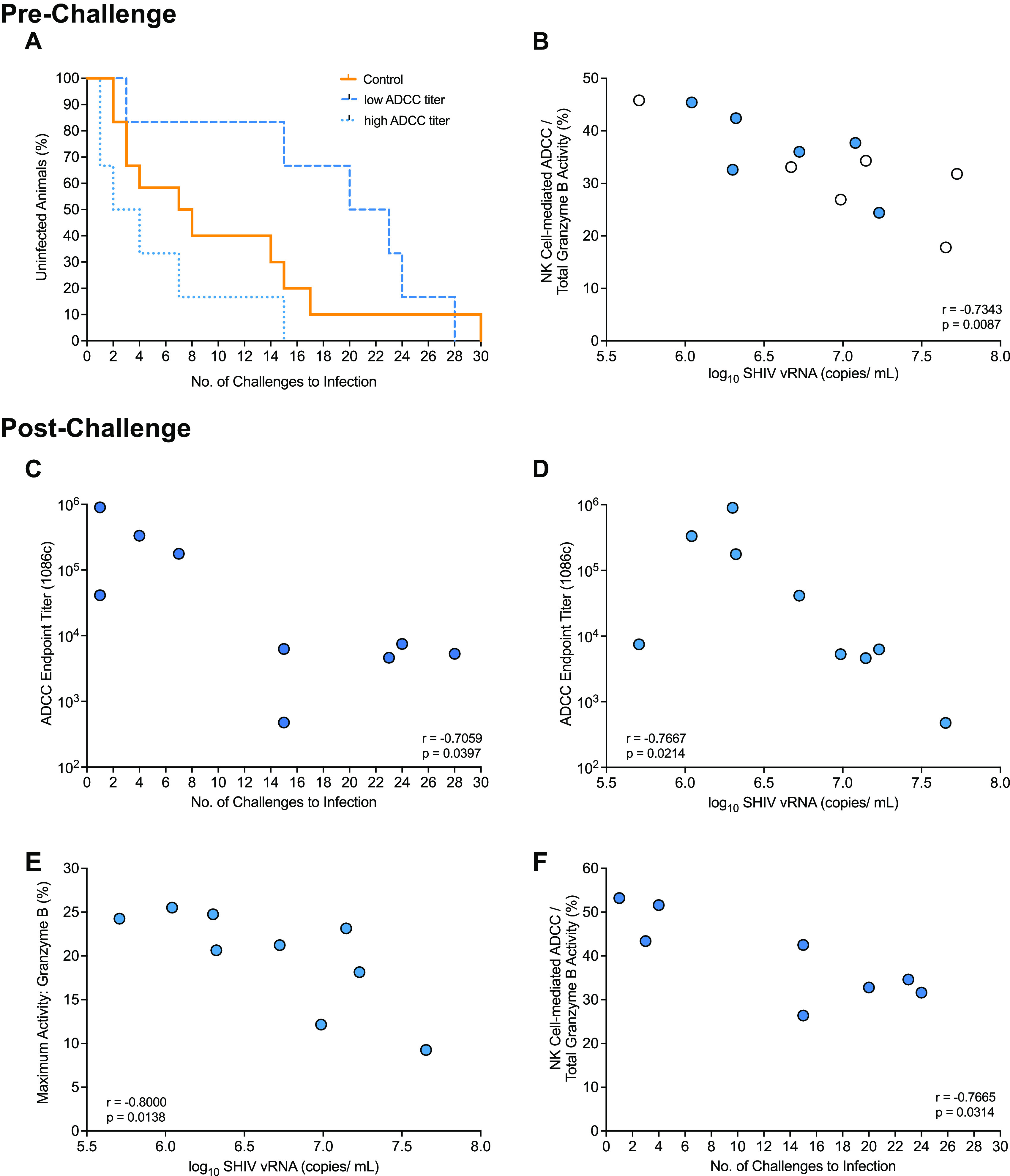
Correlation between 1086.c Env-specific antibody responses and challenge outcome. (A) Kaplan-Meier plot to demonstrate the relationship between ADCC endpoint titers and number of challenges required for infection when vaccinated animals are categorized as having a low (*n* = 6) or high (*n* = 6) ADCC titer based on the median ADCC endpoint titer of 10^5^ in comparison to control animals (*n* = 12). Mantel-Cox log rank test was applied to determine differences in the risk of infection between groups. (B) Graph of the Spearman rank correlation between ADCC endpoint titers and number of challenges required for infection of each vaccinated animal (*n* = 12). Animals with ADCC titers below or above the median ADCC endpoint titer are indicated by empty or filled blue circles, respectively. Panels C to F illustrate correlation between Env-specific antibody responses postinfection and challenge outcome. Note that limited plasma volumes did not allow us to assess postinfection antibody responses in all 12 vaccinated animals. Panels C and D show the negative correlation of endpoint 1086.c ADCC titers at week 1 postinfection with the number of challenges required for infection (C) and peak viremia (D) for 9 vaccinated animals. Panel E illustrates that higher ADCC activity at week 1 postinfection was associated with reduced peak viremia (*n* = 9). (F) In animals that required fewer challenge exposures to infection, the percentage of NK cell-mediated ADCC activity at week 4 postinfection was higher than in animals that required a greater number of exposures to infection (*n* = 8).

A more detailed assessment of Fc-mediated effector functions of Env-specific plasma IgG revealed that vaccine virus-specific ADCC activity was associated with fewer challenges required for infection (*r* = −0.761 and unadjusted *P* = 0.0054), but this inverse correlation did not reach statistical significance after adjustment for multiple-parameter analysis (adjusted *P* = 0.0984) (Table 2). This trend was most apparent when vaccinated infant RM were stratified by median ADCC titer (2.99 × 10^5^). The results suggested that animals with ADCC titers below the median required more SHIV exposures to become infected than animals with ADCC titers above the median (Fig. 8A). However, the probability to infection was not different between control animals, vaccinated animals with ADCC titers below the median, and vaccinated animals with ADCC titers above the median (log rank test with exact *P* of 0.06). Consistent with comparable median ADCC titers for 1086.c and 1157ipd3N4 Env, 1157ipd3N4 Env-specific ADCC titers trended toward a negative correlation with the number of challenges required for infection, although this trend was not substantiated after adjusting for FDR (Table 2; *r* = −0.568, unadjusted *P* = 0.0580, and FDR-adjusted *P* = 0.4548). Although ADCC activity, as assessed by maximum granzyme B production, was not correlated with the number of exposures required to achieve infection, the higher the percentage of NK cell-mediated ADCC activity, the lower was peak viremia (*r* = −0.734, unadjusted *P* = 0.0087, and FDR-adjusted *P* = 0.1163) (Table 3; Fig. 8B). In contrast, ADCP function at the time of challenge initiation appeared to be positively correlated with peak viremia (*r* = 0.706, unadjusted *P* = 0.0129, and FDR-adjusted *P* = 0.1163) (Table 3).

Based on these results, and to further substantiate a potential role for Env-specific antibody responses in challenge outcome, we tested whether recall 1086.c Env-specific antibody responses at weeks 1 and 4 postinfection were associated with challenge outcome. Animals that became infected after more numbers of challenges had higher 1086.c Env-specific plasma IgG concentrations at 1 week postinfection (Table 4). Higher 1086.c Env-specific ADCC endpoint titers at 1 week postinfection, as observed at week 15 prior to challenge, were associated fewer challenges required to achieve infection (Table 4; Fig. 8C). Nonetheless, ADCC titers were negatively associated with peak viremia, and this was likely related to the finding that higher ADCC activity at week 1 postinfection was correlated with lower peak viremia (Table 4; Fig. 8D and E). However, in contrast to the ADCC activity prior to challenge, at week 4 postinfection, a higher contribution of NK cell-mediated ADCC function to ADCC activity was observed in vaccinated animals that required fewer numbers of challenges to achieve infection (Table 4; Fig. 8F). These findings emphasize the importance of the balance between magnitude and quality of immune responses in challenge outcome.

**TABLE 4:** Correlation between 1086.c Env-specific recall antibody responses and challenge outcome

Challenge outcome/parameter	*n*	Spearman *r* value	Correlation p value^*a*^	FDR^*b*^ p-value	Figure
No. of exposures to infection					
Env-specific IgG					
Wk 1 postinfection	10	**−0.677** ^*c*^	**0.0366**	0.1588	Fig. S1 in the supplemental material
Wk 4 postinfection	9	−0.544	0.1356	0.4484	Fig. S1
ADCC titer					
Wk 1 postinfection	9	**−0.706**	**0.0397**	0.1588	Fig. 8C
Wk 4 postinfection	8	−0.180	0.6984	0.6984	
ADCC activity					
Wk 1 postinfection	9	−0.412	0.2702	0.4484	
Wk 4 postinfection	8	0.270	0.5579	0.6199	
NK cell-mediated ADCC					
Wk 1 postinfection	9	−0.395	0.2912	0.4484	
Wk 4 postinfection	8	**−0.766**	**0.0314**	0.1588	Fig. 8F
ADCP score					
Wk 1 postinfection	9	−0.378	0.3129	0.4484	
Wk 4 postinfection	8	−0.515	0.1982	0.4484	
Peak viremia					
Env-specific IgG					
Wk 1 postinfection	10	−0.418	0.2325	0.4484	Fig. S1
Wk 4 postinfection	9	−0.433	0.2499	0.4484	Fig. S1
ADCC titer					
Wk 1 postinfection	9	**−0.767**	**0.0214**	0.1588	Fig. 8D
Wk 4 postinfection	8	−0.571	0.2000	0.4484	
ADCC activity					
Wk 1 postinfection	9	**−0.800**	**0.0138**	0.1588	Fig. 8E
Wk 4 postinfection	8	−0.321	0.4976	0.5892	
NK cell-mediated ADCC					
Wk 1 postinfection	9	−0.367	0.3362	0.4484	
Wk 4 postinfection	8	−0.405	0.3268	0.4484	
ADCP score					
Wk 1 postinfection	9	−0.200	0.6134	0.6457	
Wk 4 postinfection	8	0.286	0.5008	0.5892	

a Exact *p*-value to test whether the correlation appears to be significantly different from 0.

b FDR adjustment for multiple comparisons for the sets of tests specified in Materials and Methods for the statistical analysis of antibody correlates of protection.

c Bold values indicate Spearman rank correlations with *r* > -0.6 and unadjusted *p* < 0.05.

10.1128/mSphere.00839-21.1 FIG S1 1086.c Env-specific antibody responses at week 1 and week 4 postinfection. (A) Env-specific plasma IgG concentrations in control (week 1, *n* = 9; week 4, *n* = 11) and vaccinated (week 1, *n* = 10; week 4, *n* = 9) animals. (B) ADCC endpoint titers in control (week 1, *n* = 11; week 4, *n* = 11) and vaccinated (week 1, *n* = 9; week 4, *n* = 8) animals. (C) ADCC activity in control (week 1, *n* = 8; week 4, *n* = 10) and vaccinated (week 1, *n* = 9; week 4, *n* = 8) animals. The graph at the very right shows the percentage of NK cell-mediated activity in vaccinated animals. (D) ADCP score in control (week 1, *n* = 9; week 4, *n* = 11) and vaccinated (week 1, *n* = 9; week 4, *n* = 8) animals. (E) IgG binding to Env expressed on target cells, measured by ICABA, in control (week 1, *n* = 9; week 4, *n* = 10) and vaccinated (week 1, *n* = 9; week 4, *n* = 8) animals. Each symbol represents an individual animal. Limited plasma volumes prevented the testing of samples from all animals in each assay. Horizontal lines in each graph represent the medians. Download FIG S1, EPS file, 0.3 MB. Copyright © 2022 Curtis et al. 2022 Curtis et al. https://creativecommons.org/licenses/by/4.0/ This content is distributed under the terms of the Creative Commons Attribution 4.0 International license.

## DISCUSSION

According to the UNAIDS 2021 estimates, in 2020, every day more than 400 children became infected with HIV (1). Therefore, despite increasing access to ART, vaccine development remains an urgent task to prevent new pediatric HIV infections. The current study tested the efficacy of an MVA-Env plus Env protein with 3M-052-SE adjuvant vaccine regimen combined with an ChAdOx.1.tconsSIVma239-Gag, Pol prime, MVA.tconsSIVma239-Gag, Pol boost regimen that had been optimized to maximize Env-specific antibody responses with Fc-mediated effector function (33–35, 42) in infant RM. Several previous HIV vaccine studies in NHPs had found a correlation between reduced infection or control of viral replication and vaccine-induced antibodies mediating ADCC (28, 43–45) and/or ADCP and antibody-dependent neutrophil phagocytosis (25–27). However, despite the induction of robust Env-specific antibodies with Fc-mediated effector functions, infant RM receiving the above-described vaccine regimen were not protected against oral SHIV infection. There was also no evidence of virus control, a clinically important secondary readout of vaccine efficacy pertaining to less severe disease outcomes and reduced HIV transmission risk (46).

The reasons for lack of efficacy are likely multifold. We used a challenge virus with an Env that was heterologous to the vaccine immunogen and started challenges shortly (3 weeks) after the last vaccine immunization to closely mimic consistent, real-world exposure of infants breastfed by HIV-infected women. It is possible that some residual activation in response to immunization was still lingering. We (40, 41) and others (47–50) had previously reported that T cell activation can contribute to an enhanced risk of infection with HIV, SIV, or SHIV. Although we observed higher frequencies of TNF-α-positive peripheral blood CD4^+^ T cells at the time of the first challenge in vaccinated compared to control animals, T cell activation was not correlated with the number of SHIV challenges required for infection.

In our studies leading up to the current vaccine study (33–35, 41, 42), we had focused on the optimization of Fc-mediated Env-specific IgG responses. Our vaccine regimen was not designed to induce tier 2 neutralizing antibodies that are thought to be essential in the protection against SHIV infection in RM (51). We had further reasoned that the inclusion of chimpanzee adenovirus (ChAd)- and MVA-vectored vaccines expressing SIV Gag and Pol would induce antiviral T cell responses capable of controlling virus replication at the entry site. However, SIV Gag-specific T cell responses elicited by the ChAd- and MVA-vectored vaccines were of relatively low magnitude, and neither peripheral blood mononuclear cell (PBMC) nor lymph node CD4^+^ and CD8^+^ T cell responses at week 14 correlated with the number of challenges to achieve infection or with peak viremia.

Our challenge outcome results are consistent with those of other infant and adult NHP studies that failed to demonstrate efficacy against SIV or SHIV infection by antibodies with Fc-mediated effector function only (51–53), and human HIV vaccine trials following and building on the results of the RV144 trial did not observe a reduced HIV infection risk. In the RV144 trial, protective ADCC function was primarily associated with V1V2- and C1-specific antibodies (54, 55). Our vaccine regimen, however, appears to be biased toward the induction of V3 over V1V2-specific and C5- versus C1-specific epitopes (35). Furthermore, plasma IgG responses specific to the V1V2 region of the vaccine 1086.c Env and of the challenge virus SHIV1157(QNE)Y173H were of lower avidity than the relevant gp120-specific IgG. Limited plasma volumes prevented us from assessing ADCC and ADCP activity of epitope-specific antibodies in addition to gp120-specific antibodies in the current study. In future studies, more targeted, epitope-specific analyses—including impact of glycosylation and epitope conformation—may prove beneficial in the interpretation of vaccine outcomes (55, 56).

It is also important to note that the detailed analysis of RV144 results found that trial participants with medium levels of ADCC activity had reduced infection risk compared to that of participants with low levels of ADCC activity, while there was no such difference found when comparing those with high and low vaccine-induced ADCC responses (see supplement to reference 54). In the current study, 1086.c-specific plasma antibodies with ADCC activity could be detected at a median endpoint titer of 1:10^5^ at the time of challenge initiation. Paradoxically, although individual animals with high ADCC titers (above the group median) were as likely to acquire infection as their control counterparts, 1086.c ADCC titers below the median appeared to be associated with more challenges to achieve infection, although this difference did not reach statistical significance. One potential explanation for this observation is an *in vivo* prozone, a phenomenon when high antibody in the presence of limiting antigen results in smaller immune complexes that cluster fewer Fc domain receptors on the surface of target cells and limit killing activity (57, 58). A prozone effect was also described in an early HIV infection study (59) in which plasma IgG concentrations above 10 μg/mL inhibited NK cell lysis. The importance of NK cells in ADCC-mediated protection by Env-specific antibodies was underlined by our findings that NK cell-mediated ADCC activity prior to challenge at week 15 was associated with reduced peak viremia. These data, and data from passive immunization of mice (60), suggest that there may be an optimal level, with lower and upper limits, at which nonneutralizing antibodies are most effective. However, what these levels are in the context of different exposures and how they potentially impact challenge outcome are not yet known.

Similarly, it is difficult to discern from the current literature whether there is an optimal ADCP score. Despite several studies suggesting a correlation between ADCP function and reduced HIV risk in human adults (61, 62) or SHIV infection in adult RM (25), ADCP activity elicited by the vaccine tested in the current study was not correlated with protection against oral SHIV1157(QNE)Y375H infection in infant RM. While the simple comparison of various antibody functions across different vaccine regimens, age groups, and challenge regimens is likely flawed, and different assay conditions may further impact data, the results of our study imply that the magnitude of ADCC or ADCP activity alone is not a reliable predictor of vaccine efficacy. More research is needed to assess the impact of antibody subtype, effector cell and specific Fc receptors mediating the specific functions on vaccine efficacy in preclinical NHP studies (63), and how these findings translate to humans (64). Such findings would likely result in improved *in vitro* assays to measure antibody function and thereby enhance the predictive value of these assays for vaccine efficacy assessment. Highly relevant for pediatric studies, age-dependent differences in immune function of effector cells are not considered. There are numerous studies documenting that NK cells and monocytes exhibit reduced functional capacity, including ADCC (65) and phagocytosis, in infants compared to adults (see reviews in references [Bibr B66][Bibr B67][Bibr B70]). Few studies have examined the expression of FcRγI, FcRγII, and FcRγIII on infant NK cells, monocytes, and neutrophils (71, 72). Therefore, in future studies, we will expand the analysis of vaccine-induced B and T cell responses and also determine whether and how pediatric HIV vaccine regimens impact innate immune cells and their functions.

In summary, while the prechallenge immunogenicity data demonstrated high magnitude effector antibody functions previously tied to some HIV vaccine efficacy, our results imply that Env-specific ADCC and ADCP responses induced by this candidate vaccine regimen were not sufficient to prevent infection with oral tier 2 SHIV1157(QNE)Y375H in infant RM. Therefore, future studies of interventions to protect infants against HIV acquisition through breastfeeding should focus on improving the breadth of the antibody response, namely, the induction of bNAbs or passive administration of combinations of long-acting HIV bNAbs, as well as overcoming the relative paucity of cell-mediated immunity induced by current vaccine platforms in early life.

## MATERIALS AND METHODS

### Animals and sample collection.

Twenty-four infant rhesus macaques (RM) were nursery reared and housed in pairs at the California National Primate Research Center (Davis, CA). All animal procedures were approved by the UC Davis Institutional Animal Care and Use Committee. The study strictly adhered to the guidelines outlined in the *Guide for the Care and Use of Laboratory Animals* (73) by the National Resource Council. Peripheral blood was collected by venipuncture into EDTA-treated vacutainers and processed as described previously (74). Peripheral lymph node biopsy specimens were collected at week 14 prior to initiation of oral challenges at week 15 as described previously (33). All experimental manipulations were performed under ketamine anesthesia (10 mg/kg of body weight) administered by the intramuscular (i.m.) route.

### Vaccines.

The infants in the present study were randomly divided into 2 groups of 12 (Table 1; Fig. 1). At week 0, infant RM assigned to the vaccine arm were primed i.m. with 2 × 10^8^ PFU of MVA-HIV 1086.c Env construct (in a volume of 0.25 mL divided over the left and right biceps) (35) and 15 μg of 1086Δ7 gp120 K160N protein mixed with 3M-052 adjuvant in stable emulsion (3M-052-SE) (34, 35) at a total dose volume of 0.5 mL, divided over the left and right quadriceps. The HIV Env 1086.c gp120-expressing MVA construct was produced as detailed elsewhere (75). In addition, infant vaccinees received 5 × 10^10^ viral particles of chimpanzee adenovirus (ChAdOx1.tSIVconsv239)-SIV Gag/Pol (0.25 mL i.m. divided over the left and right gluteus) at week 0. Infants in the vaccine cohort received two successive i.m. booster immunizations with 1086.c gp120 protein in 3M-052-SE and MVA-HIV Env (both were the same dose as the priming immunization) and 2 × 10^8^ PFU of MVA.tSIVconsv239 (Gag/Pol-expressing vector) in 0.25 mL, divided over the left and right biceps) at weeks 6 and 12 (35). The ChAdOx1.tSIVconsv239 and MVA.tSIVconsv239 vectors were kindly provided by Tomáš Hanke (Oxford University, Oxford, UK) to promote the induction of SIV-specific T cell responses. Control infants received an empty MVA vector at weeks 0, 6, and 12 (Fig. 1).

### SHIV-1157(QNE)Y173H challenge of vaccinated and control macaques.

At week 15, 3 weeks after the last immunization, infant macaques were orally exposed once weekly to tier 2 SHIV-1157(QNE)Y173H, a derivative of the CCR5-tropic clade C SHIV-1157ipd3N4 (28), which was kindly provided by Sampa Santra (Harvard University, Boston, MA). The virus stock corresponded to 3.7 × 10^9^ copies/mL and had a 50% tissue culture infective dose (TCID_50_) of 4.88 × 10^8^/mL in TZM-bl cells (28, 76). SHIV-1157(QNE)Y173H (referred to here as SHIV) was selected for its high sequence homology to the 1086.c V1V2 region (28). Virus was administered as a 1:1,000 dilution of virus stock in 1 mL of sucrose-containing RPMI 1640 medium in a needleless syringe (77). Infants were considered to be systemically infected following two consecutive PCR-positive values (see below). After 13 challenges of 1:1,000, uninfected infants (*n* = 11) received an increased dose of 1:100. Following 7 challenges with 1:100-diluted virus, the viral challenge was increased to a 1:10 dilution in the remaining uninfected animals (*n* = 4). Two infants (RM19 and RM10) remained negative and became infected after challenge with a 1:2 dilution of virus stock or undiluted virus, respectively (Table 1). Approximately 12 weeks post-SHIV infection, animals were euthanized.

### SHIV RNA quantification.

Weekly quantitative analysis of SHIV RNA in plasma began on week 16 as previously described (74). Briefly, RNA was manually extracted from limited plasma volumes and assayed by reverse transcription-PCR (RT-PCR) with a limit of detection of 15 copies/mL. Data are reported as the number of SHIV RNA copy equivalents per milliliter of EDTA plasma.

### Measurement of plasma HIV Env-specific IgG by ELISA.

HIV Env-specific antibody concentrations in plasma were determined by enzyme-linked immunosorbent assay (ELISA) (33). Microtiter plates were coated with 1086Δ7 gp120K160N (3 μg/mL) overnight at 4°C and blocked with phosphate-buffered saline (PBS) plus 4% whey, 15% normal goat serum, and 0.5% Tween 20. Serially diluted plasma was added to the plate following extensive washing. IgG antibodies were detected with peroxidase-labeled anti-monkey IgG (Southern Biotech), followed by tetramethylbenzidine (TMB; KPL) and stop solution. Absorbance was read at 450 nm immediately after addition of the stop solution. The rhesusized CD4 binding site monoclonal antibody B12R1 was used as a standard (78). The concentration of HIV Env-specific IgG was calculated using a five-parameter fit curve relative to the standard using SoftMax Pro 6.3 software (Molecular Devices). To account for nonspecific binding, the positivity cutoff was selected as the concentration corresponding to 3 times the optical density (OD) of blank wells.

### Measurement of Env-specific antibodies by binding antibody multiplex assay (BAMA).

Salivary IgG and IgA and plasma IgA antibodies to gp120 were measured using a customized multiplex assay with 1086.cΔ7 gp120-conjugated fluorescent magnetic beads as previously described (33). Prior to performing IgA assays, specimens were depleted of IgG using protein G Sepharose (GE Healthcare) as described previously (79). Concentrations of gp120-specific antibodies in saliva were normalized relative to the total IgA or IgG concentrations, which were measured by ELISA. Results for saliva are presented as specific activity (nanograms of anti-gp120 IgA or IgG antibody per microgram of total IgA or IgG, respectively).

### Antibody avidity.

The avidity of IgG antibodies to 1086.cΔ7 gp120, 1086.C V1V2, gp70 consensus C V3 (33), 1157ipd3N4 gp120, and 1157(QNE)Y173H V1V2 was determined using purified total plasma IgG and surface plasmon resonance (SPR) using a Biacore 4000 instrument as described previously (33). The relative avidity score equals the binding response divided by the dissociation rate constant.

### ADCC.

Antibody-dependent cellular cytotoxicity (ADCC) activity was measured as previously reported (42). Briefly, CCR5^+^ CEM.NK^R^ T cells (AIDS Reagent Program) were coated with 1086.c or SHIV-1157ipd3N4 gp120 protein. ADCC activity was determined by the GranToxiLux (GTL) assay as described previously (33, 42, 80). Fourfold serial plasma dilutions beginning at 1:100 were incubated with target cells and human PBMCs from a cryopreserved leukapheresis sample of an HIV-seronegative donor with the 158F/V genotype for FcγRIIIa after thawing and overnight rest (80–82). ADCC function is reported as endpoint titers determined by interpolation of plasma dilutions that intercepted the positive cutoff and as the maximum proportion of target cells positive for active granzyme B (maximum activity). To determine the contribution of NK cells to ADCC activity, we applied area scaling analysis as described previously (37).

### Infected cell antibody binding assay (ICABA).

Plasma antibody binding to HIV-1 Env expressed on surfaces of infected cells was measured using an infected cell binding assay as previously described (28, 83). Briefly, CEM.NKR_CCR5_ cells were mock infected or infected with a replication-competent infectious molecular clone virus encoding 1086.c Env (84) for 48 to 72 h. Cells were then cultured in the presence of diluted plasma samples from study infants. Cells were subsequently stained with a viability marker, anti-CD4 antibody (clone OKT4; eBioscience), fixed, and permeabilized prior to staining with a fluorescein isothiocyanate (FITC)-conjugated goat anti-rhesus IgG (H+L) polyclonal antibody (Southern Biotech). Data represent the frequency of cells positive for IgG binding to Env for postvaccination samples compared to the prevaccination sample. Values were normalized by subtraction of the frequency of positive cells observed for cells stained with secondary antibody alone and mock-infected cells.

### ADCP.

Antibody-dependent cellular phagocytosis (ADCP) assay was performed as previously described (85, 86). HIV Env 1086.c K160N gp120 protein was produced in-house by transfection of 293T cells. For ADCP, the HIV Env 1086.c K160N gp120 protein was conjugated to biotin using a fast type A biotin conjugation kit (Abcam) and then captured on avidin-labeled fluorescent beads (NeutrAvidin; Invitrogen). To form immune complexes with Env-expressing beads, plasma (1:50 dilution), positive antibody controls (HIVIG, RIVIG, and VRC01), or irrelevant antibody control (influenza virus-specific monoclonal antibody CH65) was incubated with antigen-conjugated beads at 37°C for 2 h. All monoclonal antibody controls were used at a concentration of 25 μg/mL. Immune complexes were then subjected to spinoculation at 1,200 × *g* in the presence of a human-derived monocyte line, THP-1 (ATCC TIB-201), for 1 h at 4°C. Following spinoculation, bead-conjugated antigens and cells were incubated at 37°C to allow for phagocytosis to occur. After 1 h of incubation, THP-1 cells were fixed with 2% paraformaldehyde (Sigma) and fluorescence of the cells was assessed by flow cytometry (BD; Fortessa). A “no antibody” control consisting of PBS supplemented with 0.1% bovine serum albumin (1× PBS plus 0.1% BSA) was used to determine the background phagocytosis activity. Phagocytosis scores were calculated by multiplying the mean fluorescence intensity (MFI) and frequency of bead-positive cells and dividing by the MFI and frequency of bead-positive cells in the PBS/BSA control. All plasma samples were tested in two independent assays, and the average phagocytic scores from these 2 independent assays was reported.

### Neutralizing antibody characterization.

Neutralizing antibodies were tested as previously reported (87). Briefly, serum was heat inactivated for 1 h at 56°C, diluted in cell culture medium, and preincubated with HIV-1 pseudotyped virus (88) for 1 h. Following preincubation, TZM-bl cells were added and incubated for 48 h. Cells were subsequently lysed and luciferase activity was determined using a luminometer and BriteLite Plus reagent (PerkinElmer). Neutralization titers were defined as the serum dilution which reduced relative light units by 50% relative to control wells after background subtraction.

### Flow cytometric analysis.

**(i) T cell activation.** PBMCs were isolated from blood as previously described (74). A total of 10^6^ PBMCs were stained with surface antibodies listed in Table 5 at room temperature for 20 min in the dark. Cells were treated with Cytofix/Cytoperm (BD Biosciences) per the manufacturer’s protocol and subsequently stained with intracellular marker antibodies (Table 5) in the same manner. Stained cells were fixed with 1% paraformaldehyde (Electron Microscopy Services). A total of 300,000 events were collected using a BD LSRFortessa and analyzed using FlowJo v10.6.1.

**TABLE 5:** FACS reagent information^*a*^

Panel/marker	Type	Fluorochrome	Clone	Vendor
Activation				
Viability dye	Surface	Aqua	NA	Invitrogen
CD3	Surface	BV421	SP34-2	BD Biosciences
CD4	Surface	PerCP-Cy5.5	L200	BD Biosciences
CD8	Surface	Alexa Fluor 700	RPA-T8	BD Biosciences
CD14	Surface	BV786	M5E2	BD Biosciences
CD16	Surface	PE-CF594	3G8	BD Biosciences
CD20	Surface	APC-H7	2H7	BD Biosciences
CD69	Surface	PE-Cy7	FN50	BD Biosciences
CD195	Surface	PE	3A9	BD Biosciences
HLA-DR	Surface	BV711	G46-6	BD Biosciences
PD-1	Surface	APC	eBioJ105	eBioscience
Ki-67	Intracellular	FITC	B56	BD Biosciences
TNF-α	Intracellular	BV650	Mab11	BD Biosciences
Antigen-specific T cells				
Viability dye	Surface	Aqua	NA	Invitrogen
CD3	Surface	APC-Cy7	SP34-2	BD Biosciences
CD4	Surface	PE-CF594	L200	BD Biosciences
CD8	Surface	BV786	RPA-T8	BD Biosciences
CD45RA	Surface	V450	5H9	BD Biosciences
CCR7	Surface	PE-Cy7	3D12	BD Biosciences
IL-2	Intracellular	PerCP-Cy5.5	MQ1-17H12	BD Biosciences
IL-17	Intracellular	PE	eBio64CAP17	BD Biosciences
IFN-γ	Intracellular	Alexa Fluor 700	B27	BD Biosciences
TNF-α	Intracellular	APC	Mab11	BD Biosciences

a Abbreviations: FACS, fluorescence-activated cell sorting; NA, not applicable; PerCP, peridinin chlorophyll protein; PE, phycoerythrin; APC, allophycocyanin.

**(ii) SIV Gag-specific T cell responses.** SIV Gag-specific T cell responses were determined as described previously (89). Briefly, 2 × 10^6^ cells were cultured in RPMI 1640 medium supplemented with glutamine, 10% heat-inactivated fetal bovine serum (FBS), and penicillin/streptomycin and stimulated with a vehicle (dimethyl sulfoxide [DMSO]), 0.5× cell stimulation cocktail (eBioscience), or 5 μg of SIV p27Gag peptide pool (NIH AIDS Reagent Program) for 6 h, with 1× brefeldin A present after the first hour. Cells were stained with antibodies (Table 5) and analyzed as described above.

### Statistical analyses.

Statistical tests were performed using R version 3.6.2.

### (i) Probability of infection.

Kaplan-Meier curves and log rank tests with exact *P* values were used to assess differences between the two groups in the probability of infection at any challenge dose. We presented curves and tested for differences in the probability of infection at any dose. One animal missed seven weekly challenges before resuming challenges on the 1:100 dose and becoming infected on its first 1:100 dose challenge. Thus, the animal was treated as censored at its seventh challenge, (a 1:1,000 dose). We estimated the per-challenge probability of infection at each dose administered (1:1,000, 1:100, 1:10, 1:2, and 1:1) as the number of animals infected by a challenge at the dose/total number of challenges (across and within all animals) administered up to and including the week of infection at the dose. For each per-challenge probability of infection, we constructed an approximate 95% confidence interval (Wilson score interval without continuity correction) by assuming that all challenges across and within animals are independent.

### (ii) Antibody correlates of protection.

We assessed the association of Env-specific plasma IgG, salivary IgG, salivary IgA, antibody avidity, neutralizing antibodies, ADCC, infected cell binding, and ADCP at week 15 with the number of challenges required to achieve SHIV infection in vaccinated animals only (Table 2). The same antibody response parameters were assessed in association with peak viremia (Table 3). Spearman’s rank correlation coefficients were estimated to assess these associations. All correlations were tested with exact *P* values to assess whether any were significantly different from 0. To adjust for multiple comparisons, the Benjamini-Hochberg (BH) procedure was used to control the false-discovery rate (FDR). An adjustment to control the FDR at a value of 0.05 was performed for these endpoints for a total 18 parameters per infection outcome.

Furthermore, to determine the impact of memory Env-specific plasma IgG, ADCP, and ADCC responses at week 1 and week 4 postinfection on challenge outcome, we tested whether these vaccine-induced recall antibody responses were associated with the number of challenges required to achieve SHIV infection or with peak viremia. All correlations were tested with exact *P* values to assess whether any were significantly different from 0, and the BH procedure was used to adjust for multiple comparisons. An adjustment to control the FDR at a value of 0.05 was performed for these endpoints for a total 20 parameters (Table 4).

### (iii) Cellular correlates of protection.

Wilcoxon rank sum tests with exact *P* values were used to compare the CCR5^+^ (CD195^+^), Ki-67^+^, CD69^+^, and CD279^+^ (PD1) CD4^+^ T cells at week 15 between vaccinated and control animals. An adjustment to control the FDR at an α value of 0.05 was performed for these 5 endpoints using the BH procedure.

Spearman’s rank correlation coefficients were estimated for the cohort as a whole as well as for vaccinated animals only. All correlations were tested with exact *P* values to assess whether any were significantly different from 0. The entire cohort was used to assess whether there was an association between CD4^+^ T cell activation parameters at week 15 and the number of challenges required for SHIV infection. Vaccinated animals were used to assess whether there appeared to be an association between Env-specific B cells and the number of challenges required for SHIV infection. To adjust for multiple comparisons, the BH procedure was used to control the FDR. An adjustment to control the FDR at an α value of 0.05 was performed for these prespecified correlation endpoints for a total 6 parameters (Table 2).
